# Tumor Transcriptome Reveals High Expression of IL-8 in Non-Small Cell Lung Cancer Patients with Low Pectoralis Muscle Area and Reduced Survival

**DOI:** 10.3390/cancers11091251

**Published:** 2019-08-26

**Authors:** Sarah Santiloni Cury, Diogo de Moraes, Paula Paccielli Freire, Grasieli de Oliveira, Douglas Venâncio Pereira Marques, Geysson Javier Fernandez, Maeli Dal-Pai-Silva, Érica Nishida Hasimoto, Patricia Pintor dos Reis, Silvia Regina Rogatto, Robson Francisco Carvalho

**Affiliations:** 1 Department of Morphology, Institute of Biosciences, São Paulo State University (UNESP), Botucatu 18618-689, São Paulo, Brazil; 2 Department of Surgery and Orthopedics, Faculty of Medicine, São Paulo State University (UNESP), Botucatu 18618687, São Paulo, Brazil; 3 Experimental Research Unit, Faculty of Medicine, São Paulo State University (UNESP), Botucatu 18618687, São Paulo, Brazil; 4 Department of Clinical Genetics, Vejle Hospital, Institute of Regional Health Research, University of Southern Denmark, Vejle 7100, Denmark

**Keywords:** secretome, computed tomography, Interleukin-8, tumor-derived factor, C2C12 cells, cachexia

## Abstract

Cachexia is a syndrome characterized by an ongoing loss of skeletal muscle mass associated with poor patient prognosis in non-small cell lung cancer (NSCLC). However, prognostic cachexia biomarkers in NSCLC are unknown. Here, we analyzed computed tomography (CT) images and tumor transcriptome data to identify potentially secreted cachexia biomarkers (PSCB) in NSCLC patients with low-muscularity. We integrated radiomics features (pectoralis muscle, sternum, and tenth thoracic (T10) vertebra) from CT of 89 NSCLC patients, which allowed us to identify an index for screening muscularity. Next, a tumor transcriptomic-based secretome analysis from these patients (discovery set) was evaluated to identify potential cachexia biomarkers in patients with low-muscularity. The prognostic value of these biomarkers for predicting recurrence and survival outcome was confirmed using expression data from eight lung cancer datasets (validation set). Finally, C2C12 myoblasts differentiated into myotubes were used to evaluate the ability of the selected biomarker, interleukin (IL)-8, in inducing muscle cell atrophy. We identified 75 over-expressed transcripts in patients with low-muscularity, which included *IL-6, CSF3,* and *IL-8*. Also, we identified *NCAM1*, *CNTN1*, *SCG2*, *CADM1*, *IL-8*, *NPTX1*, and *APOD* as PSCB in the tumor secretome. These PSCB were capable of distinguishing worse and better prognosis (recurrence and survival) in NSCLC patients. *IL-8* was confirmed as a predictor of worse prognosis in all validation sets. In vitro assays revealed that IL-8 promoted C2C12 myotube atrophy. Tumors from low-muscularity patients presented a set of upregulated genes encoding for secreted proteins, including pro-inflammatory cytokines that predict worse overall survival in NSCLC. Among these upregulated genes, *IL-8* expression in NSCLC tissues was associated with worse prognosis, and the recombinant IL-8 was capable of triggering atrophy in C2C12 myotubes.

## 1. Introduction

Lung cancer is the most prevalent cancer type worldwide and responsible for an estimated 1.8 million deaths, each year [1]. Most patients (~ 85%) develop non-small cell lung cancer (NSCLC) [2], which is frequently diagnosed in an advanced stage, and consequently has an unfavorable prognosis [3]. Cancer cachexia is a syndrome that affects a considerable proportion of NSCLC patients [4]. It is characterized by an ongoing loss of skeletal muscle mass (with or without loss of fat mass) that cannot be fully reversed by conventional nutritional support and is associated with significant functional impairments [5]. 

The loss of skeletal muscle mass in cancer cachexia may lead to substantial weight loss and decreased body mass index (BMI), which are associated with worse outcome in NSCLC patients [6,7,8]. Studies using computed tomography (CT) images have revealed occult muscle depletion in NSCLC patients, regardless of overall body weight [4,9]. Also, both the detection of muscle depletion or low muscle mass by CT images have been associated with shorter time to tumor progression, increased risk of chemotherapy toxicity, and shorter survival in NSCLC patients [4,9,10,11,12,13,14]. Skeletal muscle depletion detected by CT images in these patients also negatively affects their functional status and quality of life [15,16]. Indeed, CT-derived pectoralis muscle area (PMA) analysis has been already used to evaluate sarcopenia and to correlate low PMA with shorter survival and inflammation in NSCLC patients [14,17]. To our knowledge, tumor-secreted factors with the prognostic value associated with low PMA as detected by CT in NSCLC are unknown.

Several studies have highlighted that macromolecules secreted from cancer cells and cells within the tumor microenvironment (secretome), including many pro-inflammatory cytokines, act systemically leading to muscle wasting in cancer cachexia [18,19,20]. However, the secretome complexity and differences found in distinct lung cancer and cells lines [21,22,23] illustrate the need to apply global approaches, to identify tumor-specific secreted molecules associated with skeletal muscle depletion. Moreover, previous “omics” studies of cancer secretome in cachexia have focused on the analysis of cachectic conditioned media of single cancer cells lines to identify mediators of the syndrome [24,25,26]. However, in vitro systems ignore the contributions of the host–tumor microenvironment and the tumor heterogeneity as well as provide no insight into the disease progression [23]. These findings emphasize the importance of cancer cachexia studies in exploring the tumor secretome. Thus, we hypothesized that a tumor transcriptome-based secretome analysis in NSCLC patients with low-muscularity is a strategy capable of identifying prognostic biomarkers and mediators of cancer-associated muscle loss.

Herein, we analyzed a cohort of NSCLC patients with CT images, clinical findings, and tumor expression microarrays data from a previous study that decoded tumor radiomics features associated with gene expression levels [27]. For these patients, we compared the pectoralis muscle area with muscle normalizations based on different radiomics features to select an approach for screening muscularity. Next, we identified genes predicted to be secreted in patients with low-muscularity and assessed their prognostic value as tumor markers of recurrence-free survival and overall survival. Finally, we demonstrated the potential of interleukin (IL)-8 as a putative secreted marker capable of inducing atrophy in C2C12 myotubes.

## 2. Results

**Figure 1:**
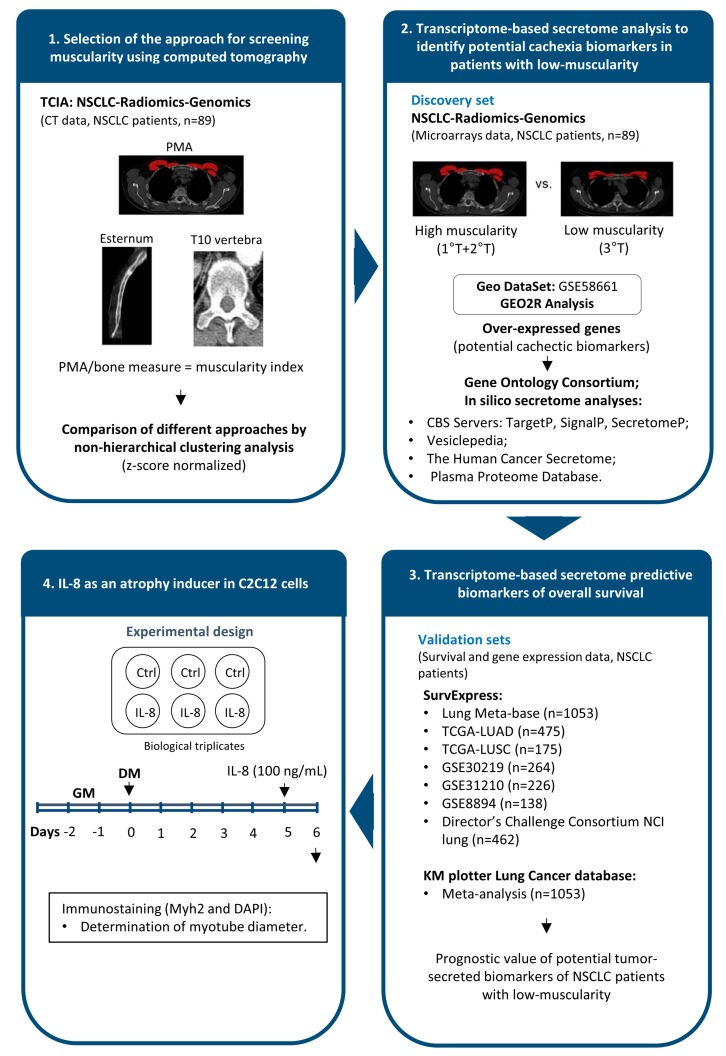
The workflow of the integrative analyses of computed tomography (CT) images and tumor transcriptome data to identify secreted cachexia biomarkers in non-small cell lung cancer (NSCLC) patients with low-muscularity. (**1**) We selected CTs from 89 patients with NSCLC from “NSCLC-Radiomics-Genomics” collection [28]. A total of 12 CTs features, including pectoralis muscle area (PMA), manubrium, and sternum body lengths, six T10 (tenth thoracic) different measures, and an anteroposterior length were used to determine an approach for screening muscularity. (**2**) This analysis revealed that PMA allows the identification of NSCLC patients with low- (third tercile, 3rd T) and high-muscularity (first and second terciles, 1st T + 2nd T). These groups were compared by using a tumor transcriptomic-based secretome analysis (discovery set; microarray data; GSE58661) to identify potential cachexia biomarkers (over-expressed genes) in patients with low-muscularity. Transcripts with increased expression were further analyzed to identify enriched terms by Gene Ontology Consortium and to predict potentially secreted proteins using secretome and microvesicle databases. (**3**) The performance of these transcripts as tumor biomarkers able to determine patients’ survival outcome was validated in multiple independent lung cancer validation sets. (**4**) C2C12 myoblasts differentiated into myotubes were used to evaluate the ability of the selected biomarker (IL-8 (interleukin-8)) in inducing atrophy. C2C12 mouse myoblasts were cultured in a growth medium (GM) for two days. Myoblasts with 80% to 90% of confluence were induced to differentiate in a differentiation medium (DM) for five days when the cells were treated with recombinant IL-8 (100 ng/mL for 24 h). TCIA: The Cancer Imaging Archive; TCGA: The Cancer Genome Atlas; LUAD: lung adenocarcinoma; LUSC: lung squamous cell carcinoma; GSE: Gene Expression Omnibus accession numbers; NCI: National Cancer Institute; KM: Kaplan Meier; n: number of patients; Ctrl: Control; Myh2: myosin heavy chain 2; DAPI: 4′,6-Diamidine-2′-phenylindole dihydrochloride nuclear staining.

The workflow of the integrative analyses of CT images and tumor transcriptome used to identify potentially secreted cachexia mediators and biomarkers in NSCLC patients with low-muscularity is depicted in Figure 1.

### 2.1. Study Population

CT images, clinical, and microarrays data of 89 NSCLC patients, with an average age of 65.2 ± 8.7 years, were included in this study. The most common NSCLC histological type was adenocarcinoma (47.2%), and 20.2% of the patients were diagnosed with advanced-stage cancers (stages III or IV). Adenocarcinoma was prevalent in women, while squamous cell carcinoma was more frequent in men. The muscle measurements revealed differences between sexes, with men and women presenting PMA of 42.5 ± 9.3 and 27 ± 6.0 cm^2^, respectively. Based on this finding, the sex-specific categorical variable was taken into consideration for further analyses. Table 1 summarizes the clinical, histopathological, and muscle measurements in this cohort of NSCLC patients. 

### 2.2. PMA Distinguishes NSCLC Patients with Low- and High-Muscularity

**Figure 2:**
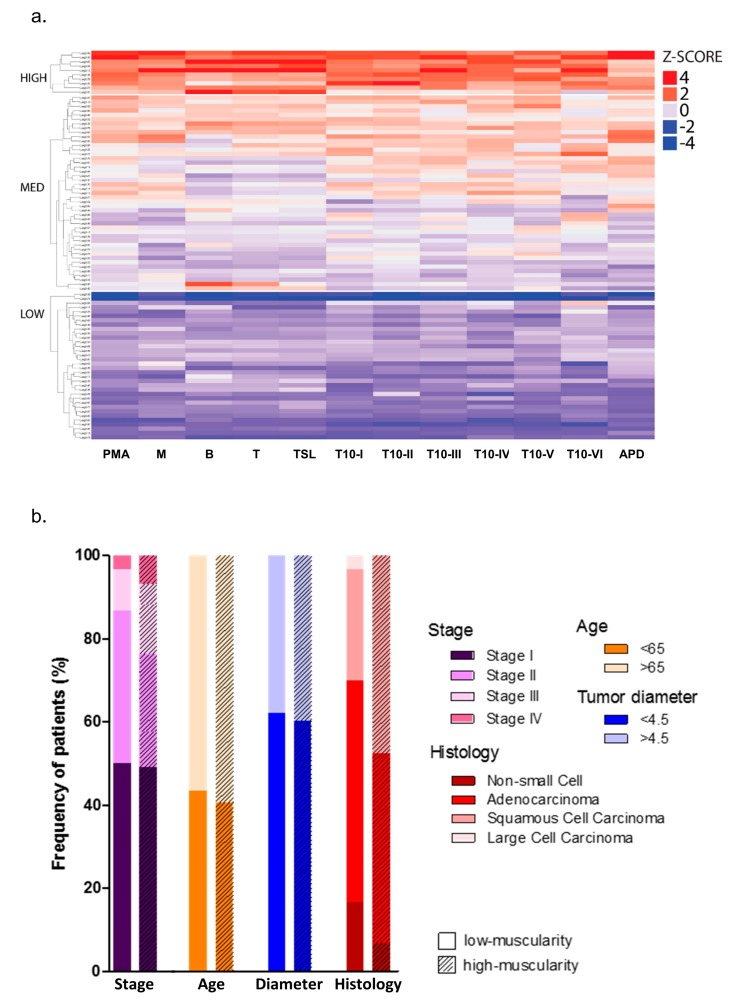
Pectoralis muscle area as an approach for screening muscularity. (**a**) Heatmap showing patients’ stratification into high-, medium-, and low-muscularity by non-hierarchical k-means clustering analysis of the pectoralis muscle area (PMA) and its normalization by eleven computed tomographies (CT) feature that includes: manubrium and sternum body lengths, six T10 (tenth thoracic) different measures, and an anteroposterior length. Manubrium length (M); sternum Body length (B); T (M+B); total sternum length (TSL); T10 body vertical length (T10-I); distance between T10 body and spinous process (T10-II); T10 body horizontal length (T10-III); distance between T10 pedicles (T10-IV); distance between T10 transverse processes (T10-V); T10 body area (T10-VI); anteroposterior distance (APD). (**b**) Bar graphs comparing the percentage of patients for clinical prognostic variables between high- and low-muscularity groups. These groups were generated based

Considering that CTs from NSCLC patients have information that goes beyond the tumor, we integrated different radiomics features to determine an approach to be used for screening muscularity (Figure S1). The non-hierarchical, unsupervised clustering analysis of the PMA and its normalization by 11 CTs features (z-score normalized) revealed a similar pattern of patients’ distribution according to all muscularity indexes. The clustering analysis also revealed three subgroups of patients according to the muscularity indexes as depicted in the dendrogram in  Figure S2a. Applying k-means analysis (k-means = 3) resulted in a cluster composed of 34 patients with low-muscularity (Figure 2a). Next, we used a descending PMA order based on gender as a sex-specific categorical variable. Finally, we segregated into terciles to generate two groups of study based on the patients’ muscularity. The low-muscularity group included patients within the third tercile, while the high-muscularity group included patients within the first and second terciles, regardless of the patient gender (Figure 2a). We highlighted that PMA could be used to select potential NSCLC low-muscularity patients; moreover, we suggested cut-offs values of PMA <32.2 cm^2^ and <21 cm^2^ (for men and women, respectively), as demonstrated by median values in the scatter dot plot in  Figure S2b. The mean PMA differed significantly between the high- and low-muscularity groups considering all patients or comparing male and female patients (Table 1). We further compared high- and low-muscularity patients with other clinical variables using patient demographic information (Figure 2b). The comparison between these groups (high- and low-muscularity) revealed that muscularity seems to be related to tumor type and tumor stage, rather than age and tumor size (Figure 2b). on the ordination of patients according to their PMA in descending order using a sex-specific categorical variable followed by segregation into terciles (high-muscularity group: 1st and 2nd terciles; low-muscularity group: 3rd tercile).

### 2.3. Patients with Low-Muscularity Upregulate Tumor Genes Previously Associated with Cachexia

**Figure 3:**
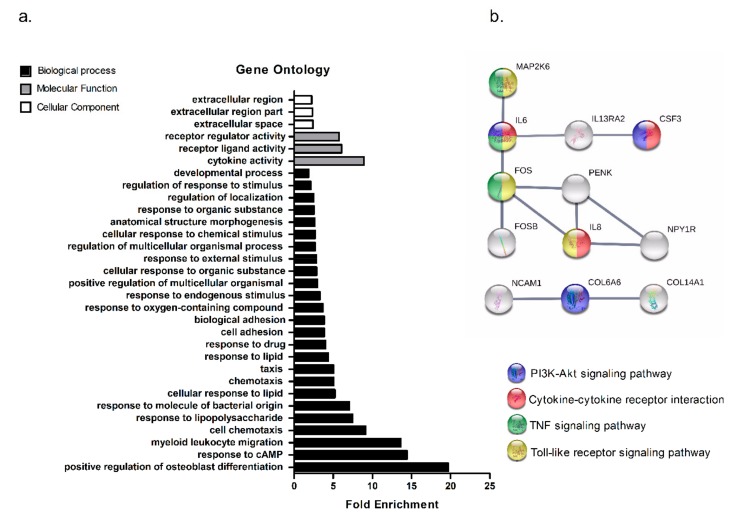
Over-expressed genes in tumors from patients with low-muscularity. (**a**) Enriched terms in gene ontology analysis of the 75 transcripts upregulated in patients with low-muscularity. (**b**) Protein-protein interaction (PPI) network of 75 upregulated transcripts in patients with low-muscularity generated by STRING (Search Tool for the Retrieval of Interacting Genes/Proteins) using a high confidence interaction score (0.700).

Considering that mediators released from cancer cells and cells within the tumor microenvironment have been associated with cachexia in lung cancers, we hypothesized that the identification of tumor deregulated genes in NSCLC patients with low-muscularity could reveal potential factors associated with cachexia. Thus, an analysis using differential gene expression between patients with low- and high-muscularity revealed 105 genes exclusively deregulated (adj. *p*-value ≤ 0.05 and fold change ≥1.5) in patients with low-muscularity, of which 75 and 30 were over- or down-expressed, respectively (Table S1). Gene ontology and Kyoto Encyclopedia of Genes and Genomes (KEGG) pathway analyses of the over-expressed transcripts highlighted cytokine activity and cytokine-receptor interaction activity as the most enriched categories in low-muscularity patients (Figure 3a). Protein-protein interaction (PPI) analysis identified the interactions among these proteins (Figure 3b), including the pro-inflammatory cytokines IL-6, IL-8, and Colony Stimulating Factor 3 (CSF3), which have been previously implicated in the development of cancer cachexia [29,30,31,32]. 

### 2.4. Secretome-Related Genes with Prognostic Value in NSCLC

**Figure 4:**
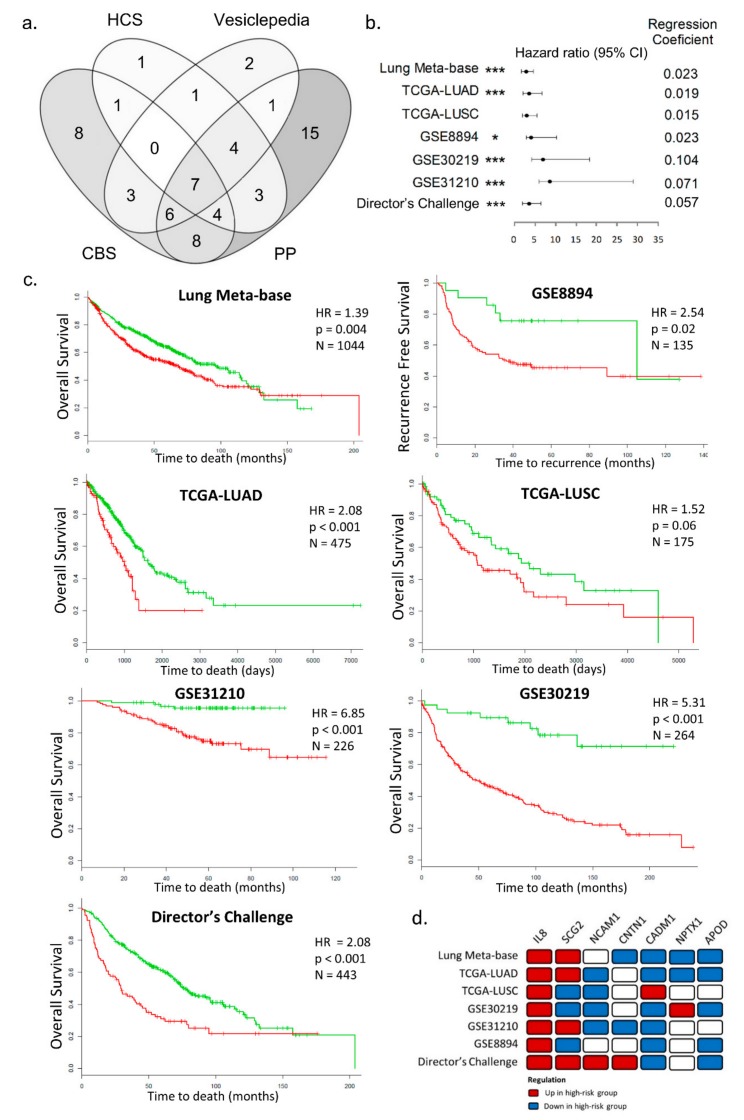
Prognostic values of the potentially secreted proteins in tumors from patients with low-muscularity. (**a**) The intersection of databases used for prediction of secreted proteins revealed seven overlapped proteins: interleukin-8 (IL-8), SCG2, NCAM1, CNTN1, CADM1, NPTX1, and APOD. (**b**) Forest plots representing the set of the seven potentially secreted biomarkers in each validation set. The horizontal axis represents confidence intervals estimated by using a Cox proportional hazards model. The asterisks represent the statistical significance in the patient survival outcome (*** *p* < 0.001 and * *p* < 0.05, log-rank *p*-value). (**c**) Kaplan-Meier plots generated in SurvExpress [33] database for the non-small cell lung cancer (NSCLC) datasets (gene expression and survival or time to recurrence): Lung Meta-base, TCGA-LUAD, TCGA-LUSC, GSE30219, GSE31210, and Director’s Challenge Consortium from National Cancer Institute (NCI). The Kaplan-Meier plot generated using the dataset GSE8894 was based on gene expression and time to recurrence data. (**d**) The direction of expression for the seven biomarkers in each validation set. HCS: Human Cancer Secretome; CBS: Servers TargetP, SecretomeP, and SignalP; PP: Plasma Proteome database; N: number of patients; HR: adjusted hazard ratio; p: log-rank *p*-value determined from univariate Cox regression analyses (green curve: low-risk group; red curve: high-risk group); TCGA: The Cancer Genome Atlas; LUAD: Lung Adenocarcinoma; LUSC: Lung Squamous Cell Carcinoma; IL-8: Interleukin-8; SCG2: Secretogranin II; NCAM1: Neural Cell Adhesion Molecule 1; CNTN1: Contactin 1; CADM1: Cell Adhesion Molecule 1; NPTX1: Neuronal pentraxin 1; APOD: Apolipoprotein D; GSE: Gene Expression Omnibus accession numbers.

We then investigated whether these 75 upregulated transcripts in the tumors from low-muscularity patients are translated into secreted proteins. The intersection of the secretome databases CBS Servers, Vesiclepedia, Human Cancer Secretome Database, and Plasma Proteome Database showed seven overlapping proteins: IL-8, Secretogranin II (SCG2), Neural Cell Adhesion Molecule 1 (NCAM1), Contactin 1 (CNTN1), Cell Adhesion Molecule 1 (CADM1), Neuronal pentraxin 1 (NPTX1), and Apolipoprotein D (APOD) (Figure 4a). The microvesicle databases revealed that the predicted proteins in Evpedia (Lipoprotein Lipase (LPL), APOD, and Collagen Type XIV Alpha 1 Chain (COL14A1)) were also identified in the Vesiclepedia dataset. However, the Exocarta did not show any of these proteins in lung cancer samples, possibly due to the limited number of exosomes studies in lung cancers deposited in this database.

The prognostic value related to the worse prognosis of IL-8, SCG2, NCAM1, CNTN1, CADM1, NPTX1, and APOD tumor transcripts were evaluated in seven lung cancer transcriptome datasets (validation set). Notably, these biomarkers were capable of distinguishing worse and better prognosis (recurrence and survival) in seven NSCLC cohorts from the SurvExpress database (Figure 4c). Interestingly, only IL-8 was found with increased expression in the high-risk group in all NSCLC validation set (Figure 4d and Figure S3).

### 2.5. High IL-8 Expression in Tumor Tissues is Associated with Poor Prognosis in NSCLC 

**Figure 5:**
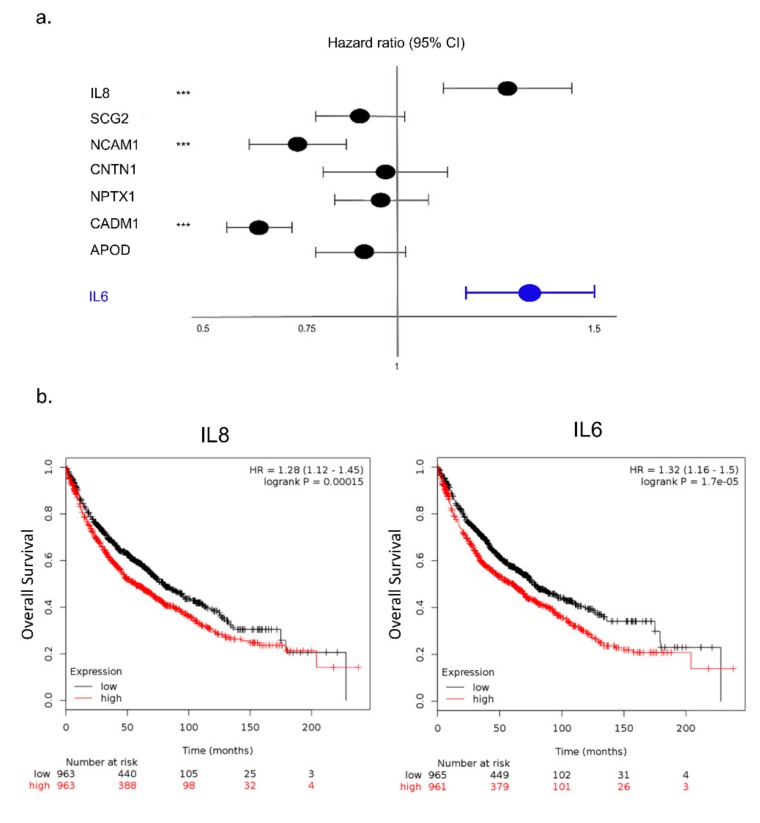
Interleukin (IL)-8 is associated with poor prognosis in non-small cell lung cancer (NSCLC). (**a**) Forest plot for each tumor biomarkers (IL-8, SCG2, NCAM1, CNTN1, CADM1, NPTX1, and APOD) in NSCLC patients from the dataset available on Kaplan-Meier (KM) plotter database. The hazard ratio (HR) with 95% confidence intervals (CI) determined by Cox proportional hazards model is represented in the horizontal axis. *** represent the statistical significance in NSCLC patient survival outcome (*p* < 0.001; log-rank *p*-value). (**b**) Kaplan-Meier overall survival curves for IL-8 or IL-6 in NSCLC patients from the dataset available on KM plotter [35] database. The resulting p-values for the log-rank test are shown. SCG2: Secretogranin II; NCAM1: Neural Cell Adhesion Molecule 1; CNTN1: Contactin 1; CADM1: Cell Adhesion Molecule 1; NPTX1: Neuronal pentraxin 1; APOD: Apolipoprotein D; IL6: Interleukin-6.

All seven potential biomarkers were individually analyzed in the Kaplan-Meier (KM) plotter server using gene expression and survival data of lung cancer patients available on the database (N = 1053), and IL-8 proved to be a strong predictor of poor survival (Figure 5a). Moreover, as IL-6 is a key regulator of muscle mass during cachexia [34] and has been associated with worse prognosis in lung cancer patients [35,36], we compared the prognostic value of IL-8 with IL-6 using KM plotter server. Notably, both IL-8 and IL-6 tumor transcripts presented similar prognostic values (IL-8: hazard ratio (HR) = 1.28, 95% confidence interval (CI) = 1.12–1.45; IL-6: HR = 1.32, 95% CI = 1.16–1.5). These results demonstrate the upregulation of IL-8 as a new biomarker associated with poor prognosis in lung cancer patients.

The 75 over-expressed transcripts were carefully evaluated in patients with low-muscularity in KM plotter to detect additional potential cachexia biomarkers associated with poor prognosis in lung cancer patients. Nine genes (*IL-6, IL-8, IL-1R2, CEMIP, CLEC4E, FCGR3B, HAL, MAP2K6*, and *KIF1A*) were validated as over-expressed in patients with worse overall survival (Figure S4). Importantly, IL-6, IL-8, IL-1R2, CEMIP, FCGR3B, and KIF1A were predicted as a potentially secreted protein in at least two secretome databases (Table S2). Collectively, these results emphasize that IL-8 is highly expressed in tumors from NSCLC patients with low-muscularity and is associated with poor prognosis in this cancer type. 

### 2.6. IL-8 Treatment Induces In Vitro Myotube Atrophy

**Figure 6:**
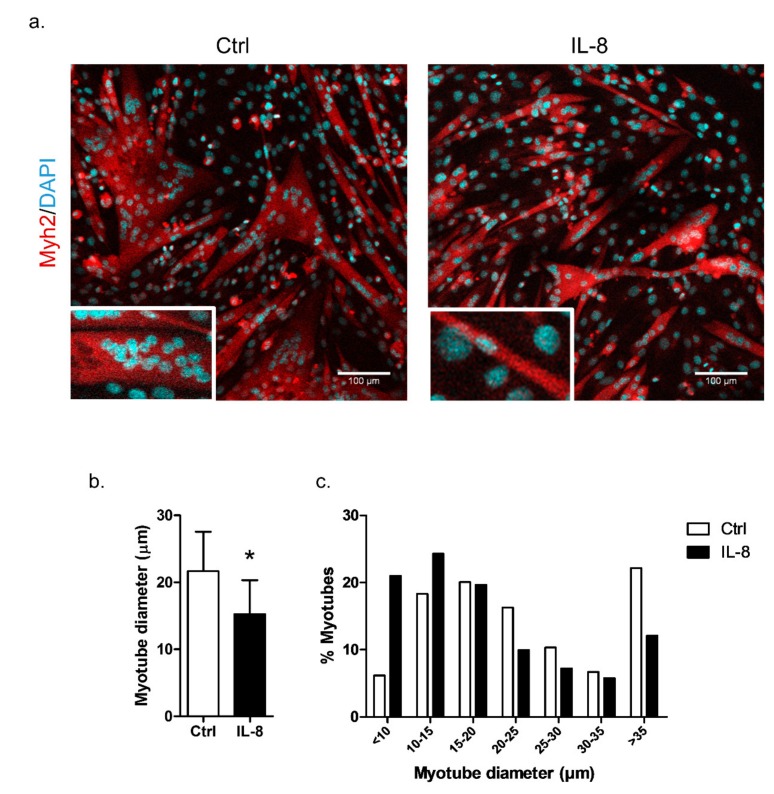
IL-8 (interleukin-8) induces atrophy in C2C12 myotubes. (**a**) Immunofluorescence of C2C12 myotubes treated with recombinant IL-8 (100 ng/mL for 24 h) immunostained for Myh2 (myosin heavy chain 2) (red) and the nuclei were counterstained with DAPI (4′,6-Diamidine-2′-phenylindole dihydrochloride). White rectangles represent a myotube bigger than 35 µm (Ctrl) or smaller than 10 µm (IL-8). Scale bars, 100 μm. (**b**) Myotube diameter (µm) quantification using ImageJ software (ImageJ, WI, USA). (**c**) Determination of the frequency of myotubes according to the diameter classes. The data represent the mean ± standard deviation from three independent experiments. The statistical significance was analyzed using the Student’s *t*-test. * *p*-value < 0.05. Ctrl: control myotubes; IL-8: myotubes treated with recombinant interleukin 8.

The ability of IL-8 in inducing muscle atrophy was evaluated by treating C2C12 myotubes with different concentrations of this cytokine (10, 100, and 1000 ng/mL). The myotubes treated with the supraphysiological dose of 100 ng/mL presented a significant decrease in diameter compared to the control group after 24 h (Figure S5). The C2C12 myotubes treated with 100 ng/mL of IL-8 for 24 h were evaluated by Myh2 (myosin heavy chain 2) immunostaining, which confirmed the significant decrease in myotubes diameter (Figure 6a,b). Myotubes treated with IL-8 also presented a higher number of myotubes with <10 µm of diameter compared to the control group. Conversely, a higher number of controls myotubes with >35 µm of diameter compared to those myotubes treated with IL-8 was observed (Figure 6c).

## 3. Discussion

Using a tumor transcriptome-based secretome analysis in NSCLC patients with low-muscularity, we aimed to identify potential cancer biomarkers of prognostic value and mediators of cancer-associated muscle loss. This strategy revealed increased expression levels of cachexia-related genes predicted to be secreted in NSCLC from patients with lower PMA. These genes were further associated with shorter recurrence-free survival and decreased overall survival in different validation sets of patients with NSCLC. Importantly, increased expression levels of *IL-8* were detected in the high-risk group in all NSCLC validation sets, and IL-8 was sufficient to trigger atrophy in C2C12 myotubes. 

Muscle depletion or low muscle mass in NSCLC patients identified by CT images has been extensively associated with poor outcome [4,9,10,11,12,13,15,16]. Previous studies using the same methodology to ours—the objective assessment of the PMA on CT scans—reported lower PMA associated with worse overall survival in NSCLC patients or cases with chronic obstructive pulmonary disease, despite normalization for BMI and performance status [14,37]. Teigen et al. reported that the PMA divided by height (used to standardize for body size) is a powerful predictor of outcome after left ventricular assist device implantation [38]. Unfortunately, the height in our cohort of PMA CT-based analysis was not available. However, the high quality of these CT images previously allowed the identification of new tumor radiomics features with prognostic value in NSCLC patients [27]. Thus, we hypothesized that the comparison of the PMA with muscle normalizations based on different radiomics features aiming the standardization for body size could reveal new approaches for screening muscularity in NSCLC patients. Interestingly, PMA distinguished NSCLC patients with low- and high-muscularity in all muscle normalizations tested. Considering that CTs images of lung cancer patients are preferentially performed in the thoracic region, our data additionally confirm that PMA is a feasible measurement easily applied to the clinical practice to distinguish NSCLC patients with different muscularity. 

Although a large range of changes in body composition has been associated with tumor-derived factors, including many pro-inflammatory cytokines [18,19,20,39], only few NSCLC studies associated CT-derived body composition with systemic inflammatory response [40,41]. These studies showed that lower muscularity was associated with systemic inflammatory response (IL-6, C-reactive protein, and albumin blood levels, and neutrophil-to-lymphocyte ratio). However, the specific tumor-derived factors that induce muscle loss in NSCLC patients are still unknown. Using the tumor transcriptome analysis of NSCLC patients with low-muscularity, we found 105 deregulated genes, of which 75 were upregulated and 30 downregulated. The functional enrichment analysis revealed upregulated genes related to cytokine activity (*CSF3, IL-8, IL-6, BMP6, SCG2, CCL8, BMP2*) and extracellular space (*CSF3, FLRT2, IL-8, PLA2G3, IL-6, ATP1B1, COL14A1, LPL, HBB, ADAMTS4*). These results suggest that tumor of patients with low-muscularity possibly secrete cachexia-associated factors.

The in silico analysis confirmed that a set of over-expressed genes were translated into proteins presented in plasma or secretome of NSCLC patients. Seven of these predicted proteins (NCAM1, CNTN1, SCG2, CADM1, IL-8, NPTX1, and APOD) were identified in five databases (SignalP 4.1, SecretomeP 2.0, Vesiclepedia, Human Cancer Secretome, and Plasma Proteome), giving support to their relevance in NSCLC. Although not all NSCLC patients with low-muscularity were cachectic, the tumor gene expression profile identified molecules, such as *IL-6* and *IL-8*, consistently linked to inflammation and cancer cachexia pathogenesis [29,30,31,42,43,44,45,46,47,48]. The low muscle mass detected by CT images can occur in the absence of systemic inflammation in other malignancies, such as colorectal cancer, but the proportion of patients with low-muscularity is substantially greater in the presence of systemic inflammation [49]. In cases where the inflammation coexists with low muscle mass, the prognosis is especially poor [50]. Taken together, we identified a specific set of upregulated genes coding for secreted proteins that may constitute potential mediators of muscle loss in NSCLC.

Based on the fact that circulating levels of tumor-derived factors were correlated with cachexia development and predicted outcome in cancer [29,30,31,42,43,44,45,46,47,48], we also investigated the predictive potential of seven transcripts (*NCAM1*, *CNTN1*, *SCG2*, *CADM1*, *IL-8*, *NPTX1*, and *APOD*). All of them were associated with shorter overall survival and recurrence-free survival for the predicted high-risk groups in the NSCLC validation set. However, only *IL-8* was over-expressed in the high-risk group in all cohorts of our NSCLC validation set. We further confirmed that high *IL-8* expression level in tumor tissue is a strong predictive biomarker significantly associated with worse survival (validation cohort of 1053 NSCLC patients). In agreement with our results, IL-8 expression in tumor tissues was recently associated with cachectic status and outcome in pancreatic cancer; cachectic patients with high IL-8 expression in tumor tissues had shorter overall survival or disease-free survival [31]. Importantly, these authors also showed that IL-8 expression level in tumor specimen paired with a serum sample from the same patients was associated with tumor size. 

We demonstrated that IL-8 directly induced myotube atrophy, reinforcing its potential as a new mediator of cancer cachexia. Muscle wasting in cancer cachexia has been attributed to the combinatorial action of mediators from host and tumor microenvironment [18,19,20,39]. Also, tumor expression and serum levels of IL-8 have been associated with muscle wasting in patients with different tumor types [29,31,42,43,44,45,46]. The potential direct effect of IL-8 in inducing muscle cell atrophy is still unknown. In this study, we provide evidence that IL-8 is a biomarker of worse prognosis that has the potential to define the cachectic state in NSCLC patients

The main strength of the present investigation is the identification of potential tumor-derived mediators of muscle wasting in patients with low-muscularity, which have prognostic value in NSCLC. However, our study is based on the reuse of transcriptomic and clinical data, which results in limitations that can be pointed out. Firstly, the validation of the findings at protein levels in NSCLC patients with low-muscularity would be a strategy to define the cachexia blood biomarkers useful for clinical routine. Secondly, our survival analyses were restricted to the validation set; the survival information was not available in our discovery dataset. Finally, since the *IL-8* gene is not present in the rodent genome, the atrophy phenotype observed in mice myotubes was likely induced by orthologue receptors to the human IL-8 [51]. In agreement with our study, Gerber et al. reported that IL-8 protein expression was significantly associated with tumor-free body weight and skeletal muscle weight in a human pancreatic cancer xenograft mouse model [52]. Further studies are needed to elucidate the mechanisms of action of IL-8 in human muscle cells.

## 4. Materials and Methods 

### 4.1. Datasets

CT images and clinical data were downloaded from The Cancer Imaging Archive (TCIA, http://cancerimagingarchive.net/) database [53]. The dataset (NSCLC-Radiomics-Genomics collection) [28] contains information from 89 NSCLC adult patients treated at MAASTRO Clinic, The Netherlands, as previously published [27]. TCIA data are anonymized, and the institutional ethical review board approval is not needed [54]. CT images were taken on diagnosis, and the patients were treated with surgical procedure. Clinical data (age, gender, diagnosis, tumor stage), CT images, and tumor microarrays data are available for all 89 patients. The NSCLC-Radiomics-Genomics microarrays data is available on Gene Expression Omnibus (GEO, http://www.ncbi.nlm.nih.gov/geo; microarrays dataset GSE58661) [27].

### 4.2. CT Imaging Analyses

The CT collection “NSCLC-Radiomics-Genomics” on TCIA database present CT images with radiomics features that can be used as noninvasive prognostic or predictive biomarkers [27]. This collection is also the most appropriate due to the homogeneity of the CT images. The pectoralis muscle was analyzed on a single axial slice of the image. This region was selected by a single trained physician (ENH) who identified the aortic arch and then selected the first image just above the arch. The cross-sectional area (cm^2^) of bilateral major and minor pectoralis muscles was measured by two independent examiners, using Slice-O-Matic software (v.5.0; Tomovision, Montreal, Quebec, CA). Muscles were manually traced using the Region of Interest (ROI) tool by summing the appropriate pixels determined by CT Hounsfield unit (HU) for skeletal muscle (range −29 HU to 150 HU). The borders of the pectoralis muscles were corrected manually when necessary, as previously described [14,37,55]. The pectoralis muscle area (PMA) was calculated by adding up the four muscles area. To test the reproducibility of this analysis, an interobserver coefficient of variation was determined by comparing the results of the analyses conducted by the two observers. The mean of this coefficient of variation was 8.1%.

We also compared the PMA with muscle normalizations based on different radiomics features, as previously described [56,57,58], to test different approaches for screening muscularity in NSCLC patients. For this purpose, the pectoralis muscle area was also normalized by the following sternum measurements: 1) manubrium length; 2) sternum body length; 3) total manubrium and sternum body lengths; 4) distance between the beginning of manubrium and the end of sternum body measured in 90° (not considering the xiphoid process) (Figure S1a). Different T10 (tenth thoracic) vertebrae measurements were also tested for muscle normalizations: 1) horizontal length of T10 body; 2) vertical length of T10 body; 3) distance between T10 body and spinous process; 4) distance between transverse processes; 5) distance between pedicles; 6) T10 body area. We also analyzed the body cross-section anteroposterior diameter (APD) at the tenth thoracic vertebra (T10) level to normalize the muscle area (Figure S1b). The bone images were selected in the cross-section where the bones appeared at a higher extent and dimension. The measurements were performed at the tenth thoracic vertebra (T10), which is a common region for all patients in this CT collection. Skeletal muscle index (or muscularity) was defined as the PMA divided by each bone or body measure (mentioned above) squared (cm^2^/cm^2^). The measurements generated were z-score normalized and submitted to a non-hierarchical k-means clustering analysis using Bioconductor Package Complex Heatmap (v 3.5) in Rstudio software (RStudio, Inc, MA, USA; http://www.rstudio.org/).

### 4.3. Gene Expression Analysis

Tumor gene expression analysis was performed by comparing low- and high-muscularity patients using the GEO2R tool (http://www.ncbi.nlm.nih.gov/geo/geo2r/) [59]. The adjusted *p*-values (adj. p) were applied using Benjamini and Hochberg false discovery rate (FDR) method by default. The cut-off criteria to define differential expression were adj. *p* < 0.05 and |Fold Change (FC)|>1.5. 

### 4.4. Gene Ontology Enrichment Analysis 

Gene ontology (GO) functional enrichment analysis was performed to identify the overrepresented GO categories of differential expressed genes using Gene Ontology Consortium database (http://geneontology.org/) [59]. The GO categories with p-value and FDR <0.05 were considered significant.

### 4.5. Protein-Protein Interactions (PPI) Networks 

PPI networks were generated using the Search Tool for the Retrieval of Interacting Genes/Proteins (STRING) tool [60,61] (http://string-db.org/). We considered experiments, database, co-expression, neighborhood, and co-occurrence as active interaction sources. The minimum required interaction score was 0.700 (high confidence), and the disconnected nodes in the network were hidden for display simplifications. The PPI enrichment *p*-value indicates the statistical significance provided by STRING.

### 4.6. In Silico Identification of Secreted Proteins

The over-expressed genes in the tumor of patients with low-muscularity were filtered for genes encoding secreted proteins or proteins presented in microvesicles based on a pipeline of seven tools: SignalP 4.1 [62], SecretomeP 2.0 [63], ExoCarta [64], TargetP 1.1 [65], Human Cancer Secretome (HCS) [66], Vesiclepedia [67], and Evpedia [68]. Firstly, we accessed the UniProtKB database to obtain amino acid sequences of proteins in FASTA format [69]. These data were used in the prediction servers SignalP, TargetP, and SecretomeP at CBS portal (http://www.cbs.dtu.dk/services/). SignalP 4.1 server was used to identify classical secretory proteins (presenting signal peptide and D-value >0.45). Proteins without signal peptide were evaluated in the SecretomeP 2.0 server to determine non-classical secreted proteins, using the cut-off for a neural network (NN) score >0.6. TargetP 1.1 server was used to selectively collect proteins involved in secretory pathways and exclude mitochondrial proteins [65]. These potentially secreted proteins were also investigated in lung cancer using the tools ExoCarta, HCS, Vesiclepedia, and Evpedia. Finally, the Plasma Proteome Database was consulted to identify human plasma proteins and their isoforms potentially encoded by the over-expressed genes from low-muscularity patients [70]. The tumor over-expressed genes, detected by all eight prediction tools, were next used to assess their prognostic performance in predicting overall survival and time to recurrence in multiple NSCLC independent datasets (validation set). 

### 4.7. Prognostic Performance of Secretory Genes in Predicting NSCLC Outcome 

SurvExpress [33] database (http://bioinformatica.mty.itesm.mx/SurvExpress) was used to assess the effect of differentially expressed genes on survival (datasets: Lung Meta-base, TCGA-LUAD (The Cancer Genome Atlas–Lung adenocarcinoma) and LUSC (The Cancer Genome Atlas–Lung squamous cell carcinoma) [71], GSE30219 [72], GSE31210 [73,74], and the Director’s Challenge Consortium NCI [75]) and time to recurrence (dataset: GSE8894 [76]) of NSCLC patients. This tool allowed us to assess the expression of secretory genes in tumor tissues and their association with the survival or time to recurrence by Cox Proportional Hazard regression according to the risk groups estimated by an optimization algorithm. The prognostic value of the secretory genes in predicting survival was further determined in 1053 NSCLC patients using Kaplan-Meier Plotter—KM plotter [77]. Here, gene expression was specifically associated with survival and time to recurrence (worse prognosis) due to the lack of other clinical characteristics available in the databases. The datasets included in all survival analysis present other clinical variables, such as age, gender, histology, and stage, which were not discriminated here. 

### 4.8. Functional Assay Using the C2C12 Cell Culture 

C2C12 mouse myoblasts (ATCC^®^ CRL-1772™) were cultured in Dulbecco’s modified Eagle’s medium (DMEM, Thermo Fisher Scientific, Waltham, MA, USA) with 1% Penicillin-Streptomycin (Thermo Fisher Scientific, Waltham, MA, USA) and 10% fetal bovine serum (FBS, Thermo Fisher Scientific, Waltham, MA, USA) at 37 °C and 5% CO2 for growth and expansion. After reaching a confluence of 80–90%, the myoblasts were induced to differentiate in DMEM serum-free supplemented with 1% Penicillin-Streptomycin for five days. Human recombinant IL-8 (10, 100, or 1000 ng/mL; Abcam, Branford, CT, USA) was added to a new differentiation medium for 24 h. All experiments were conducted using three independent replicates per group. Control myotubes (Ctrl) received a sterile water solution containing bovine serum albumin 0.1%, the same solution used to dilute IL-8.

### 4.9. Immunofluorescence Assay

C2C12 myotubes cultured in 6-well plates were fixed in 4% paraformaldehyde for 15 min, washed with phosphate-buffered saline (PBS) and 0.1% TritonX-100 (Sigma, St. Louis, Missouri, USA), and blocked with 3% bovine serum albumin (BSA), 1% glycine, 8% fetal bovine serum in PBS and 0.1% TritonX-100 for 1 h at room temperature. Subsequently, the cells were incubated with primary (Myh2, 1:600 dilution, M7523, Sigma, St. Louis, MO, USA) antibody overnight at 4 °C. After washing, the cells were incubated with secondary antibody at the same concentration of the primary antibody (1 h at room temperature) and counterstained with 4′,6-diamidino-2-phenylindole—DAPI (ProLong Gold Antifade Mountant with DAPI, Thermo Fisher Scientific, Waltham, MA, USA). All images were acquired using scanning confocal microscope Fluoview FV10i (Olympus, Tokyo, Japan). The myotube diameter was measured as follow: 10 fields were randomly selected and, at least, 20 myotubes were measured in each field using the NIH ImageJ software (ImageJ, WI, USA; https://imagej.nih.gov/ij/). Next, we determined the average diameter calculating the mean of the measures taken along the length of the myotube, as previously described by Rommel et al. [78]. 

### 4.10. Statistical Analysis

For the statistical analyses not previously described, we used the GraphPad Prism® (GraphPad Software, San Diego, CA, USA). Student’s t-test or Mann-Whitney U-test was applied for independent samples with normal distributed or non-parametric data, respectively. The comparison of the effect of three different IL-8 concentrations on C2C12 myotubes with the respective controls was performed using one-way ANOVA followed by Tukey test. Data are expressed as mean ± standard deviation (SD). 

## 5. Conclusions

In conclusion, our study demonstrated that PMA is a clinical and practical method to distinguish NSCLC patients with different muscularity from routinely acquired CT images. Tumors from patients with low-muscularity have a set of upregulated genes coding for secreted proteins within the tumor microenvironment, including pro-inflammatory cytokines, which predict worse overall survival in NSCLC. Among the upregulated genes, high *IL-8* expression in tumor tissues is also associated with worse prognosis in NSCLC, and recombinant IL-8 is capable of triggering atrophy in C2C12 myotubes.

## Figures and Tables

**Table 1:** Clinical findings and skeletal muscle parameters of patients with NSCLC (non-small cell lung cancer) with low- and high-muscularity defined by pectoralis muscle cross-sectional area assessed by computed tomography.

Characteristics	All	Men	Women	*p*-value *
**Number of patients**	89	60	29	
**Age**	65.2 ± 8.7	66.9 ± 7.3	61.8 ± 10.3	0.011 ^a^
**Cancer Stage (%)**				
**Early Stages (I-II)**	79.7	74.6	90	0.08 ^b^
**Advanced Stages (III-IV)**	20.2	25.4	10	
**Histological Type (%)**				
**Adenocarcinoma**	47.2	37.3	66.6	0.02 ^b^
**Squamous Cell Carcinoma**	40.4	49.2	23.3	
**Other Subtypes**	12.4	13.5	10.1	
**PMA (cm^2^)**	37.3 ± 11.1	42.5 ± 9.3	27 ± 6.0	<0.001 ^a^
**HM (N)**	59	40	19	
**LM (N)**	30	20	10	
**LM PMA (cm^2^)**	28.6 ± 6.5 ^#,c^	32.3 ± 4.3 ^#,c^	21 ± 1.4 ^#,c^	<0.001 ^a^
**HM PMA (cm^2^)**	41.7 ± 10.2	47.5 ± 6.3	30 ± 5.2	<0.001 ^a^

N: number of patients; PMA: Pectoralis Muscle Area; LM: low-muscularity patients; HM: high-muscularity patients. The data represent the mean ± standard deviation. a: Student’s *t*-test; b: Chi-squared test; c: Mann-Whitney’ U-test; * comparisons between men and women; # statistical difference between patients with low- and high-muscularity (*p* < 0.001).

## Supplementary Materials

The following are available online at https://www.mdpi.com/2072-6694/11/9/1251/s1, Figure S1: Different bone measurements used for Pectoralis muscle area normalization; Figure S2: Identification of clusters of patients according to muscularity indexes and Pectoralis Muscle Area (PMA) cut-off values; Figure S3: Heatmaps showing gene expression findings of seven potential biomarkers in low- and high-risk groups in NSCLC validation sets; Figure S4: Highly expressed mRNAs in tumor tissue associated with poor prognosis in NSCLC; Figure S5: C2C12 myotubes treated with different concentrations of recombinant IL-8 (10, 100, 1000 ng/mL); Table S1: mRNA differentially expressed in tumors of 30 NSCLC patients with low-muscularity compared with the gene expression of 59 NSCLC patients with high-muscularity; Table S2: Potentially secreted proteins identified in secretome-related databases based on the over-expressed genes in NSCLC patients with low-muscularity.
