# Supplementary material for: Tumor Transcriptome Reveals High Expression of IL-8 in Non-Small Cell Lung Cancer Patients with Low Pectoralis Muscle Area and Reduced Survival
Source: Cancers (Basel). 2019 Aug 26;11(9):1251. doi: 10.3390/cancers11091251 (PMC6769884; doi:10.3390/cancers11091251)
Supplement: Supplementary file 1 [file cancers-11-01251-s001.pdf]

Supplementary Materials

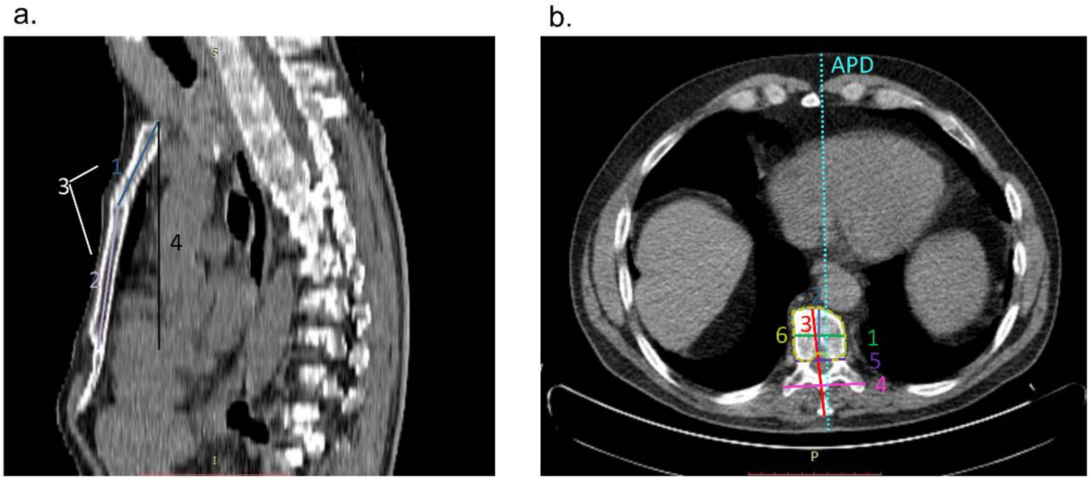

Figure S1. Different bone measurements used for Pectoralis muscle area normalization. (**a**) 1: manubrium length; 2: sternum body length; 3: sum of manubrium and sternum body lengths; 4: distance between manubrium and sternum body (without considering xiphoid process). (**b**) 1: horizontal length of T10 body; 2: vertical length of T10 body; 3: distance between T10 body and spinous process; 4: distance between transverse processes; 5: distance between pedicles; 6: T10 body area; APD: anteroposterior diameter. CT source The Cancer Imaging Archive (TCIA).

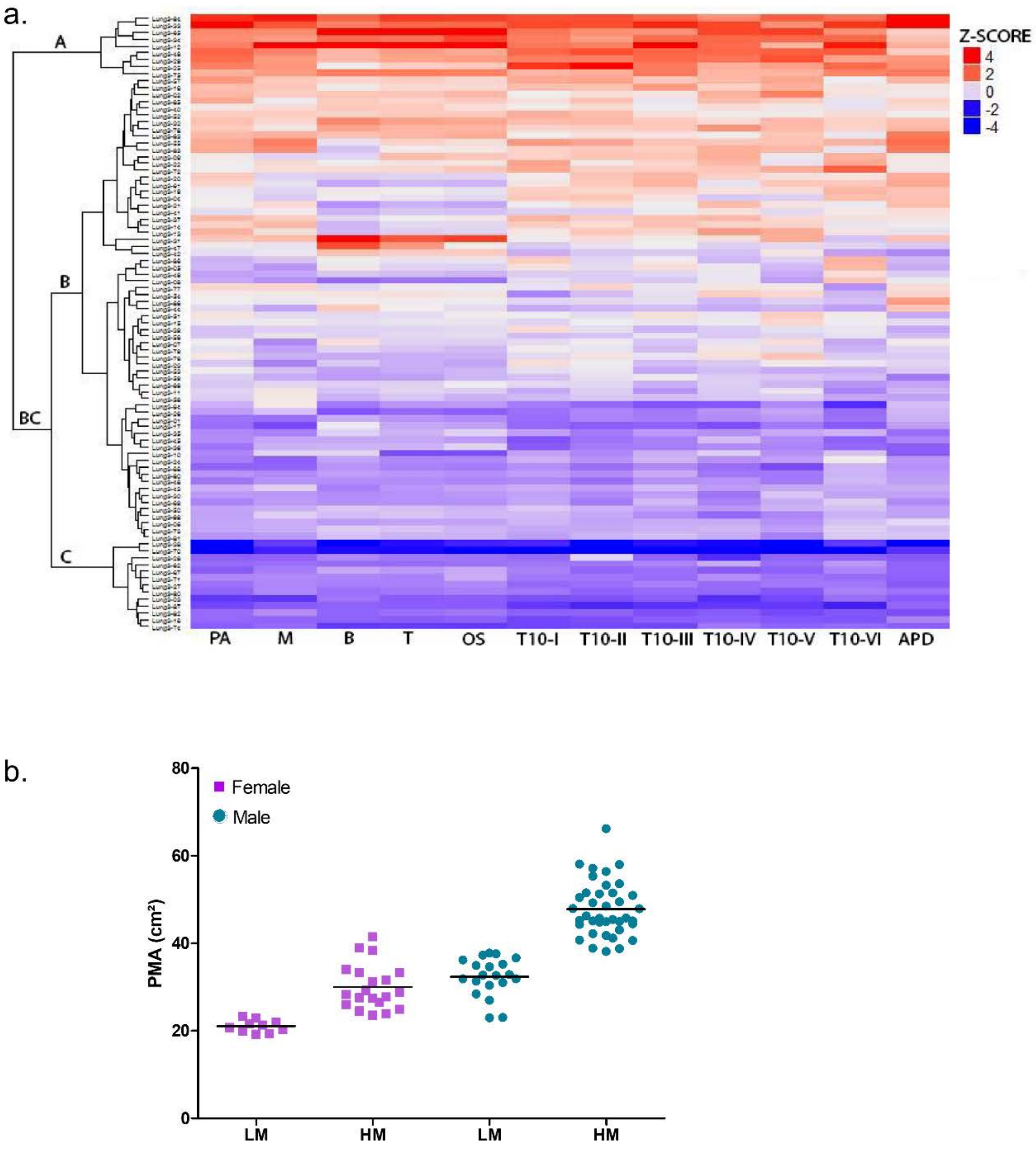

Figure S2. Identification of clusters of patients according muscularity indexes and Pectoralis Muscle Area (PMA) cut-offs values. (a) PMA clustering analysis. Non-hierarchical clustering analysis for muscularity indexes. Manubrium length (M); sternum Body length (B); T (M+B); total sternum length (OS); T10 body vertical length (T10-I); distance between T10 body and spinous process (T10-II); T10 body horizontal length (T10-III); distance between T10 pedicles (T10-IV); distance between T10 transverse processes (T10-V); T10 body area (T10-VI); anteroposterior distance (APD). (b) PMA cut-offs for women (PMA<21 cm²) and men (PMA< 32.2 cm²) as represented by median values for low-muscularity (LM) compared to high-muscularity (HM) patients.

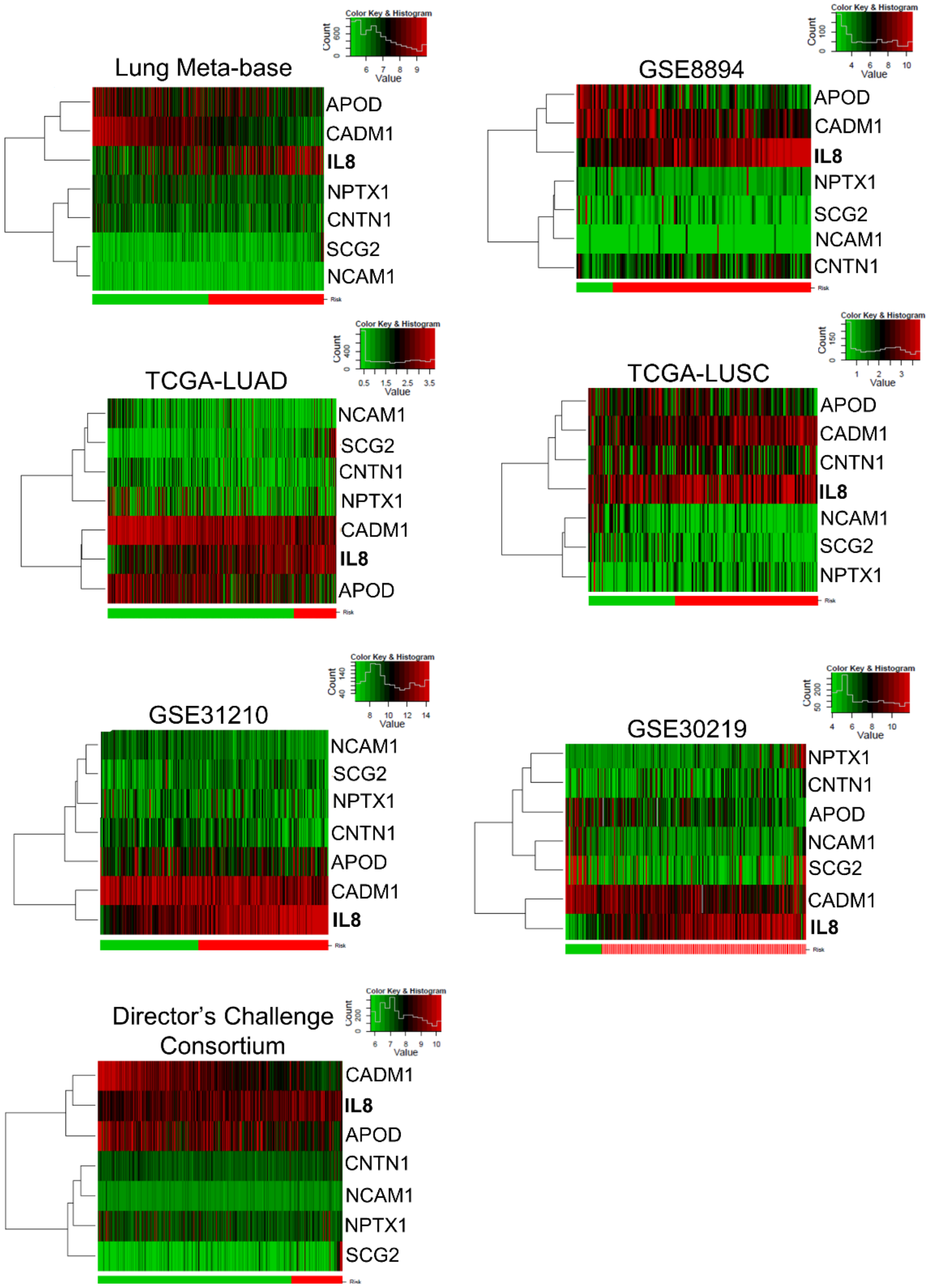

Figure S3. Heatmaps showing gene expression findings of seven potential biomarkers in low- and high-risk groups in NSCLC validation sets (SurvExpress, http://bioinformatica.mty.itesm.mx:8080/Biomatec/SurvivaX.jsp).

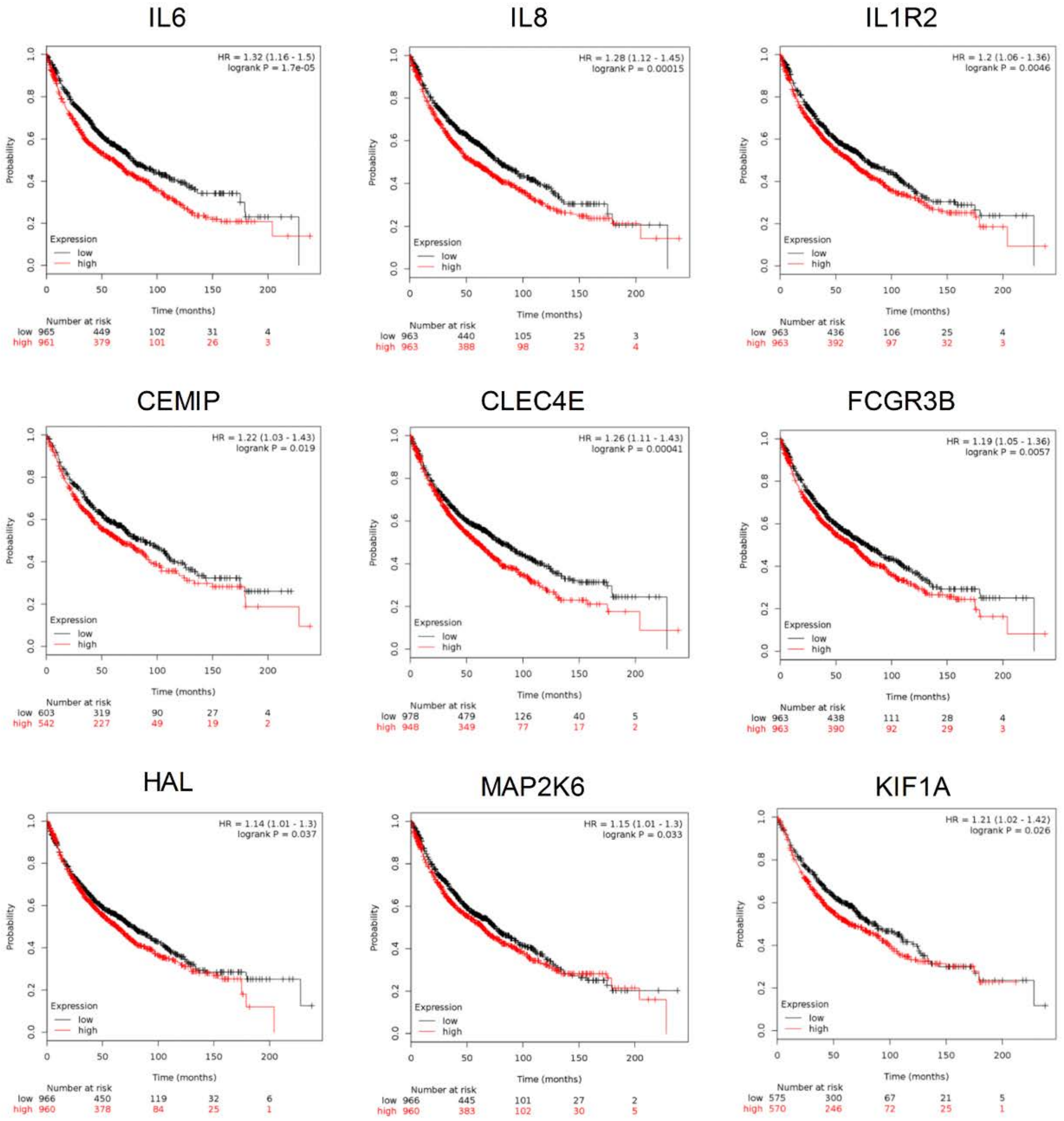

Figure S4. Highly expressed mRNAs in tumor tissue associated with poor prognosis in NSCLC. Kaplan-Meier plot generated in KMplotter database (http://www.kmplot.com/lung/) from 1053 NSCLC patients that highly express IL6, IL8, IL1R2, CEMIP, CLEC4E, FCGR3B, HAL, MAP2K6, and KIF1A in patients with worse survival.

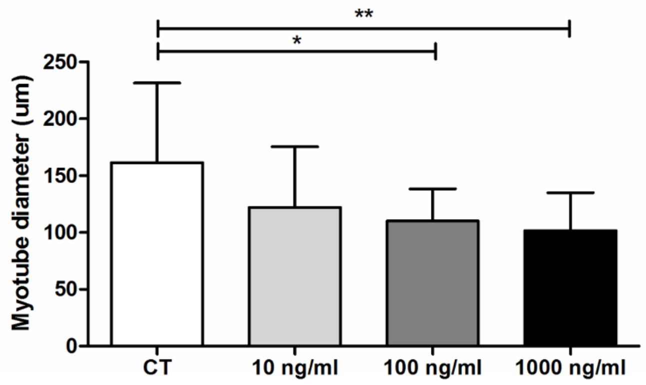

Figure S5. C2C12 myotubes treated with different concentrations of recombinant IL-8 (10, 100, 1000 ng/mL). Myotube diameter of C2C12 cells exposed to human recombinant IL-8. We captured myotubes images using phase-contrast inverted optical microscopy coupled with a digital camera. For this reason, we used these images only to analyze the biggest myotubes, in which we were able to determine the cellular limits and, consequently, to measure myotubes diameter more precisely. Experiments were performed in duplicate, and the data represent the mean ± standard deviation. Statistical analysis was performed using one-way analysis of variance test.* represents a significance of ($p < 0.05$) compared with CT; ** represents a significance of ($p < 0.01$) compared with CT. CT: control; um:micrometer.
